# Obesity and type 2 diabetes in sub-Saharan Africans – Is the burden in today’s Africa similar to African migrants in Europe? The RODAM study

**DOI:** 10.1186/s12916-016-0709-0

**Published:** 2016-10-21

**Authors:** Charles Agyemang, Karlijn Meeks, Erik Beune, Ellis Owusu-Dabo, Frank P. Mockenhaupt, Juliet Addo, Ama de Graft Aikins, Silver Bahendeka, Ina Danquah, Matthias B. Schulze, Joachim Spranger, Tom Burr, Peter Agyei-Baffour, Stephen K. Amoah, Cecilia Galbete, Peter Henneman, Kerstin Klipstein-Grobusch, Mary Nicolaou, Adebowale Adeyemo, Jan van Straalen, Liam Smeeth, Karien Stronks

**Affiliations:** 1 Department of Public Health, Academic Medical Center, University of Amsterdam, Meibergdreef 9, 1105 AZ Amsterdam, The Netherlands; 2 School of Public Health, Kwame Nkrumah University of Science and Technology, Kumasi, Ghana; 3 Institute of Tropical Medicine and International Health, Charité – University Medicine Berlin, Augustenburger Platz 1, 13353 Berlin, Germany; 4 Department of Non-communicable Disease Epidemiology, London School of Hygiene and Tropical Medicine, London, UK; 5 Regional Institute for Population Studies, University of Ghana, P.O. Box LG 96, Legon, Ghana; 6 MKPGMS-Uganda Martyrs University, Kampala, Uganda; 7 Department of Molecular Epidemiology, German Institute of Human Nutrition Potsdam-Rehbruecke, Arthur-Scheunert-Allee 114-116, 14558 Nuthetal, Germany; 8 Department of Endocrinology and Metabolism; DZHK (German Centre for Cardiovascular Research), partner site Berlin; Center for Cardiovascular Research (CCR), Charite Universitaetsmedizin Berlin, Berlin, Germany; 9 Source BioScience, Nottingham, UK; 10 ResearchGate, Invalidenstrasse 115, D-10115 Berlin, Germany; 11 Department of Clinical Genetics, Academic Medical Center, Amsterdam, The Netherlands; 12 Julius Global Health, Julius Center for Health Sciences and Primary Care, University Medical Center Utrecht, Utrecht, The Netherlands; 13 Division of Epidemiology and Biostatistics, School of Public Health, Faculty of Health Sciences, University of the Witwatersrand, Johannesburg, South Africa; 14 Center for Research on Genomics and Global Health, National Human Genome Research Institute, National Institutes of Health, 12 South Drive, MSC 5635, Bethesda, MD USA; 15 Department of Clinical Chemistry, Academic Medical Center, Amsterdam, The Netherlands

**Keywords:** Obesity, Type 2 diabetes, Migrants, Ethnic minority groups, Europe, Sub-Saharan Africa

## Abstract

**Background:**

Rising rates of obesity and type 2 diabetes (T2D) are impending major threats to the health of African populations, but the extent to which they differ between rural and urban settings in Africa and upon migration to Europe is unknown. We assessed the burden of obesity and T2D among Ghanaians living in rural and urban Ghana and Ghanaian migrants living in different European countries.

**Methods:**

A multi-centre cross-sectional study was conducted among Ghanaian adults (n = 5659) aged 25–70 years residing in rural and urban Ghana and three European cities (Amsterdam, London and Berlin). Comparisons between groups were made using prevalence ratios (PRs) with adjustments for age and education.

**Results:**

In rural Ghana, the prevalence of obesity was 1.3 % in men and 8.3 % in women. The prevalence was considerably higher in urban Ghana (men, 6.9 %; PR: 5.26, 95 % CI, 2.04–13.57; women, 33.9 %; PR: 4.11, 3.13–5.40) and even more so in Europe, especially in London (men, 21.4 %; PR: 15.04, 5.98–37.84; women, 54.2 %; PR: 6.63, 5.04–8.72). The prevalence of T2D was low at 3.6 % and 5.5 % in rural Ghanaian men and women, and increased in urban Ghanaians (men, 10.3 %; PR: 3.06; 1.73–5.40; women, 9.2 %; PR: 1.81, 1.25–2.64) and highest in Berlin (men, 15.3 %; PR: 4.47; 2.50–7.98; women, 10.2 %; PR: 2.21, 1.30–3.75). Impaired fasting glycaemia prevalence was comparatively higher only in Amsterdam, and in London, men compared with rural Ghana.

**Conclusion:**

Our study shows high risks of obesity and T2D among sub-Saharan African populations living in Europe. In Ghana, similarly high prevalence rates were seen in an urban environment, whereas in rural areas, the prevalence of obesity among women is already remarkable. Similar processes underlying the high burden of obesity and T2D following migration may also be at play in sub-Saharan Africa as a consequence of urbanisation.

**Electronic supplementary material:**

The online version of this article (doi:10.1186/s12916-016-0709-0) contains supplementary material, which is available to authorized users.

## Background

Type 2 diabetes mellitus constitutes a growing threat to human health. The International Diabetes Federation recent estimates indicate that 9 % of the global adult population (415 million people) have diabetes, with the number set to rise beyond 642 million within the next two decades [1]. The diabetes epidemic is truly a global problem with substantial variations within regions.

In high-income countries, migrant populations are particularly affected by type 2 diabetes [2]. They also develop type 2 diabetes at a younger age, and have higher associated morbidity and mortality and related complications, such as cardiovascular disease, than local European populations [3–5]. The limited data available indicate that sub-Saharan African (SSA) migrants are among those that are most affected by type 2 diabetes [2, 3]. In a recent meta-analysis, the prevalence of type 2 diabetes was nearly three times higher in populations of SSA origin than in European host populations [2]. In addition, the prevalence of type 2 diabetes differs between SSA origin populations living in different European countries. In a previous study, the prevalence of type 2 diabetes was higher among African Caribbeans living in England than in African Caribbeans living in the Netherlands [6]. This suggest that distinct environmental factors, in addition to heritable susceptibility, contribute to the development of type 2 diabetes among these populations.

The prevalence of type 2 diabetes is not only rising among migrants, but also in low- and middle-income countries such as those in SSA from where many of these populations originate [7, 8]. While type 2 diabetes seemed to be virtually absent, for example, in West Africa in the 1960s and 1980s, today it has become a major health threat particularly in urban centres [7, 8]. SSA is expected to experience the worldwide fastest increase in the number of people living with type 2 diabetes (141 %) in the next two decades [1]. The rising levels of type 2 diabetes in populations of SSA origin are a reflection of the rising levels of major risk factors such as obesity [9, 10].

The rising levels of obesity and associated type 2 diabetes among SSA origin populations is thought to be a result of transitioning of societies, and resulting changes in lifestyles, though the key specific drivers within this broad category still need to be determined [1, 9]. Migration studies provide important windows of opportunity to assess differences between migrating and non-migrating populations, and to help identify the potential factors driving the rising levels of type 2 diabetes and obesity among these populations. Such knowledge is a prerequisite for designing effective public health interventions for addressing the problem. Ideally, this requires comparing a relatively homogeneous migrant population with the source population in their country of origin in Africa. However, such data are lacking so far. Consequently, in the last two decades, studies have used migration surrogates such as multinational comparison of African descent populations living in diverse geographic environments [11, 12]. The findings, however, are difficult to interpret because of the heterogeneous nature of the African populations studied so far, and the reliance on secondary analyses of data from different studies.

The main aim of this paper was, therefore, to compare the prevalence of obesity and type 2 diabetes among Ghanaians living in rural and urban Ghana, as well as among Ghanaians living in three different European countries.

## Methods

### Study population and study design

The RODAM (acronym for Research on Obesity & Diabetes among African Migrants) study is a multi-centre cross-sectional study. The rationale, conceptual framework, design and methodology of the RODAM study have been described in detail elsewhere [13]. In brief, the study was carried out between 2012 and 2015 and it included Ghanaians aged 25–70 years living in rural and urban Ghana as well as in Amsterdam, Berlin and London. As a central feature of the RODAM study, at all study sites, a well standardised approach was used for data collection. Previous studies among African communities in Europe showed that involvement of the community leaders improves study participation [14, 15]. The RODAM study, therefore, involved the Ghanaian community leaders in all the five geographical sites.

In Ghana, two purposively chosen cities and 15 villages in the Ashanti region were used as the urban and rural recruitment sites, respectively. Participants were randomly drawn from the list of 30 enumeration areas in the Ashanti region based on the 2010 census. In Amsterdam, Ghanaian participants were randomly drawn from the Amsterdam Municipal Health register, which holds data on country of birth of citizens and their parents, thus allowing for sampling based on the Dutch standard indicator for ethnic origin. In London, there was no population register for migrant groups. Consequently, Ghanaian organisations served as the sampling frame. Lists of these organisations were obtained from the Ghanaian Embassy and the Association of Ghanaian Churches in the UK in the boroughs known to have the greatest concentration of Ghanaians. Lists of all members of their organisations were also requested. In Berlin, a list of Ghanaian individuals was provided by the registration office of the federal state of Berlin, but due to low response to written invitation based on this list, we changed to member lists of Ghanaian churches and organisations as the sampling frame. In all European sites, all selected participants from these lists were sent a written invitation combined with written information regarding the study and a response card. After a positive response, the participants were contacted by phone to schedule date and location of the interview with a trained research assistant or opt for the self-administration of the paper questionnaire or digital online version depending on the preference of the participant. Subsequent to the completion of the questionnaire, a date for physical examination was then scheduled. All the participants were instructed to fast from 10.00 pm the night prior to the physical examination.

The participation rate was 76 % in rural Ghana and 74 % in urban Ghana. In London, of those individuals that were registered in the various Ghanaian organisations and were invited, 75 % agreed and participated in the study. In Berlin, this figure was 68 %. In Amsterdam, we received a response from 67 % of those invited, either by response card or after a home visit by an ethnically-matched interviewer. Of these, 53 % agreed and participated in the study. Almost all of the Ghanaians in Europe were first generation (99 %) migrants, and the mean length of stay was generally similar across the three European sites.

### Measurements

Information on demographics, education level, medical history, treatment and lifestyle factors was obtained by questionnaire. Physical examinations were performed with validated devices according to standardised operational procedures across all study sites. Weight was measured in light clothing and without shoes with SECA 877 scales to the nearest 0.1 kg. Height was measured without shoes with a portable stadiometer (SECA 217) to the nearest 0.1 cm. Body mass index (BMI) was calculated as weight (kg) divided by height squared (m^2^). Overweight was defined as a BMI of ≥ 25 to < 30 kg/m^2^ and obesity as a BMI ≥ 30 kg/m^2^ [16]. Waist circumference was measured in centimetres at the midpoint between the lower rib and the upper margin of the iliac crest. Abdominal obesity was defined according to World Health Organization cut-offs: waist circumference > 102 cm in men and > 88 cm in women [16]. All the anthropometrics were measured twice by the same assessor and the average of the two measurements were used for analyses. Blood pressure was measured three times using a validated semi-automated device (The Microlife WatchBP home) with appropriate cuffs in a sitting position after at least 5 min rest. The mean of the last two blood pressure measurements was used in the analyses.

Fasting venous blood samples were collected by trained research assistants in all sites. All the blood samples were processed and aliquoted immediately (within 1 hour to maximum 3 hours of the vena puncture) after collection according to standard operation procedures, and then temporarily stored at the local research location at −20 °C. The separated samples were then transported to the local research centres’ laboratories, where they were checked, registered and stored at −80 °C. To avoid intra-laboratory variability, the stored blood samples from the local research centres were transported to Berlin for biochemical analyses. Fasting plasma glucose concentration was measured using an enzymatic method (hexokinase). Concentration of total cholesterol was assessed by using colorimetric test kits. All biochemical analyses were performed by using an ABX Pentra 400 chemistry analyzer (ABX Pentra; Horiba ABX, Germany). Type 2 diabetes was defined according to the World Health Organization diagnostic criteria (fasting glucose ≥ 7.0 mmol/L, or current use of medication prescribed to treat diabetes, or self-reported diabetes) [17]. Impaired fasting glycaemia (IFG) was defined as fasting glucose of between 5.6 and 6.9 mmol/L according to the American Diabetes Association definition as this threshold optimises sensitivity and specificity for predicting future diabetes [18].

### Data analysis

The characteristics of the study population were expressed as percentages with 95 % confidence intervals (CI) for categorical variables and means with 95 % CIs for continuous variables. Age-standardised prevalence rates of obesity, type 2 diabetes and IFG were calculated using the direct method, with the standards being the age distribution of the total RODAM population [19]. Prevalence ratios (PR) and their corresponding 95 % CIs were estimated by means of Poisson regression with robust variance to examine differences in prevalence between rural Ghanaians and their Ghanaian compatriots living in urban Ghana and the various European countries, respectively, with adjustment for age and education. Probabilities of type 2 diabetes and obesity by age, BMI and waist circumference were plotted using marginal effects of continuous predictors (MCP) command in STATA. All analyses were performed using STATA 14.0 (Stata Corp, College Station, Texas).

## Results

### Characteristics of the study population

Out of the 6385 Ghanaians who agreed and participated, 5659 were included in the analysis after exclusion of those who did not participate in the physical examination, those without blood samples collected and those outside the age range (Additional file 1: Figure S1). The age structure was similar in all geographical sites, although men in Amsterdam and Berlin were slightly older than in other geographical sites. Ghanaians in London were the most educated group while individuals from rural Ghana were the least educated group (Table 1). There were substantial differences in mean BMI, waist circumference, fasting glucose, total cholesterol and blood pressure among sites, with individuals in urban Ghana and Europe having higher mean levels than their counterparts in rural Ghana. Smoking prevalence was higher in Berlin and Amsterdam than in other sites.

**Table 1:** Characteristics of the population by locality and sex

	Rural Ghanaians	Urban Ghanaians	Amsterdam Ghanaians	Berlin Ghanaians	London Ghanaians
Men	(n = 405)	(n = 415)	(n = 609)	(n = 297)	(n = 410)
Age, years	46.2 (45.0–47.5)	46.5 (45.4–47.7)	48.4 (47.7–49.2)	45.8 (44.6–47.0)	46.1 (45.0–47.1)
Education level, %					
None or elementary	39.0 (34.4–43.8)	22.2 (18.4–26.4)	20.5 (17.5–23.9)	6.1 (3.9–9.4)	3.9 (2.4–6.3)
Lower secondary	36.1 (31.5–40.9)	42.4 (37.7–47.2)	40.6 (36.7–44.5)	47.8 (42.2–53.5)	24.9 (20.9–29.3)
Higher secondary	13.3 (10.4–17.0)	20.5 (16.9–24.6)	25.1 (21.8–28.7)	28.3 (23.4–33.7)	16.8 (13.5–20.8)
Tertiary education	5.7 (3.8–8.4)	9.2 (6.7–12.3)	8.2 (6.2–10.7)	17.5 (13.6–22.3)	41.0 (36.3–45.8)
Unknown	5.9 (4.0–8.7)	5.8 (6.9–8.5)	5.6 (4.0–7.7)	0.3 (0.0–2.4)	13.4 (10.4–17.7)
Length of stay in Europe, years	NA	NA	18.7 (18.0–19.4)	16.8 (15.5–18.2)	15.1 (14.1–16.1)
First generation migrants, %	NA	NA	98.6 (97.1–99.3)	99.3 (97.2–99.9)	98.6 (96.7–99.4)
Current smoking, yes, %	5.8 (3.8–8.6)	3.3 (1.9–5.6)	8.1 (6.1–10.7)	14.8 (11.2–19.3)	1.4 (1.0–3.2)
BMI, kg/m^2^	20.9 (20.6–21.2)	24.1 (23.8–24.5)	27.0 (26.7–27.3)	26.4 (26.0–26.9)	27.5 (27.1–27.9)
Waist, cm	76.8 (76.0–77.6)	84.7 (83.7–85.7)	91.1 (87.2–95.1)	91.2 (89.9–92.5)	75.3 (61.2–89.4)
Total cholesterol, mmol/L	4.2 (4.1–4.3)	5.1 (5.0–5.2)	5.1 (5.0–5.1)	5.2 (5.0–5.3)	5.0 (4.9–5.1)
Fasting glucose, mmol/L	5.1 (5.0–5.2)	5.8 (5.5–6.0)	5.7 (5.5–5.9)	5.5 (5.2–5.7)	5.3 (5.2–5.5)
Systolic BP, mmHg	123.9 (122.0–125.7)	131.0 (129.0–133.0)	138.2 (136.8–139.6)	138.9 (136.8–141.0)	136.6 (134.9–138.3)
Diastolic BP, mmHg	77.4 (76.3–78.4)	82.2 (81.0–83.5)	87.9 (87.0–88.8)	88.7 (87.4–90.0)	84.6 (83.6–85.7)
Known diabetes, %	1.2 (0.5–2.9)	7.0 ( 4.9–9.9)	11.7 (9.3–14.5)	12.8 (9.4–17.1)	7.6 (5.4–10.6)
Newly detected diabetes, %	2.0 (1.0–3.9)	3.9 (2.4–6.2)	2.1 (1.2–3.6)	2.7 (1.4–5.3)	2.2 (1.1–4.2)
Women	(n = 638)	(n = 1034)	(n = 931)	(n = 250)	(n = 670)
Age, years	46.7 (45.7–47.6)	44.7 (44.1–45.4)	45.6 (45.0–46.1)	44.7 (43.5–45.8)	47.7 (46.9–48.5)
Education level, %					
None or elementary	62.2 (58.4–65.9)	50.5 (47.5–53.6)	40.8 (37.7–44.0)	11.6 (8.2–16.2)	10.0 (8.0–12.5)
Lower secondary	26.0 (22.8–29.6)	35.9 (33.0–38.9)	30.7 (27.8–33.7)	54.0 (47.8–60.1)	28.9 (26.5–33.4)
Higher secondary	3.0 (1.9–4.6)	8.5 (7.0–10.4)	17.9 (15.5–20.5)	24.8 (19.8–30.5)	24.2 (21.1–27.6)
Tertiary education	1.9 (1.1–3.3)	2.7 (1.9–3.9)	3.8 (2.7–5.2)	7.6 (4.9–11.6)	22.1 (19.1–25.3)
Unknown	6.9 (5.2–9.2)	2.3 (1.6–3.4)	6.9 (5.4–8.7)	6.9 (5.4–8.7)	2.0 (1.0–4.7)
Length of stay in Europe, years	NA	NA	17.7 (17.2–18.2)	16.9 (15.7–18.2)	17.4 (16.5–18.3)
First generation migrants, %	NA	NA	99.5 (98.8–99.8)	99.6 (97.2–99.9)	96.9 (95.2–98.1)
Current smoking, yes, %	0.0 (0.0–0.1)	0.1 (0.0–1.0)	2.1 (1.3–3.4)	3.3 (1.6 (6.3)	0.2 (0.0–1.2)
BMI, kg/m^2^	23.7 (23.3–24.0)	28.0 (27.7–28.3)	30.3 (30.0–30.6)	29.1 (28.5–29.7)	30.9 (30.5–31.3)
Waist, cm	81.9 (78.2–85.7)	90.0 (87.6–92.4)	94.7 (92.0–97.4)	93.7 (92.3–95.1)	80.4 (69.8–91.0)
Total cholesterol, mmol/L	4.7 (4.6–4.8)	5.3 (5.2–5.3)	5.0 (4.9–5.1)	5.1 (5.0–5.3)	5.0 (4.9–5.1)
Fasting glucose, mmol/L	5.2 (5.1–5.3)	5.5 (5.4–5.7)	5.4 (5.3–5.4)	4.8 (4.7–5.0)	5.2 (5.1–5.3)
Systolic BP, mmHg	123.7 (122.0–125.5)	124.7 (123.5–125.9)	131.9 (130.8–133.0)	132.0 (129.7–134.3)	134.4 (133.1–135.7)
Diastolic BP, mmHg	76.9 (76.0–77.9)	78.3 (77.6–79.0)	82.0 (81.4–82.7)	83.6 (82.2–85.0)	82.3 (81.5–83.0)
Known diabetes, %	3.3 (2.2–5.0)	4.9 (3.8–6.4)	8.3 (6.7–10.2)	8.8 (5.9–13.0)	7.0 (5.3–9.2)
Newly detected diabetes, %	2.4 (1.4–3.9)	3.5 (2.5–4.8)	1.0 (0.5–1.8)	0.8 (0.2–3.2)	1.8 (1.0–3.1)

Values are means or percentages with corresponding 95 % confidence intervals

BP, blood pressure; BMI, body mass index; NA, not available

### Prevalence of obesity

**Fig. 1:**
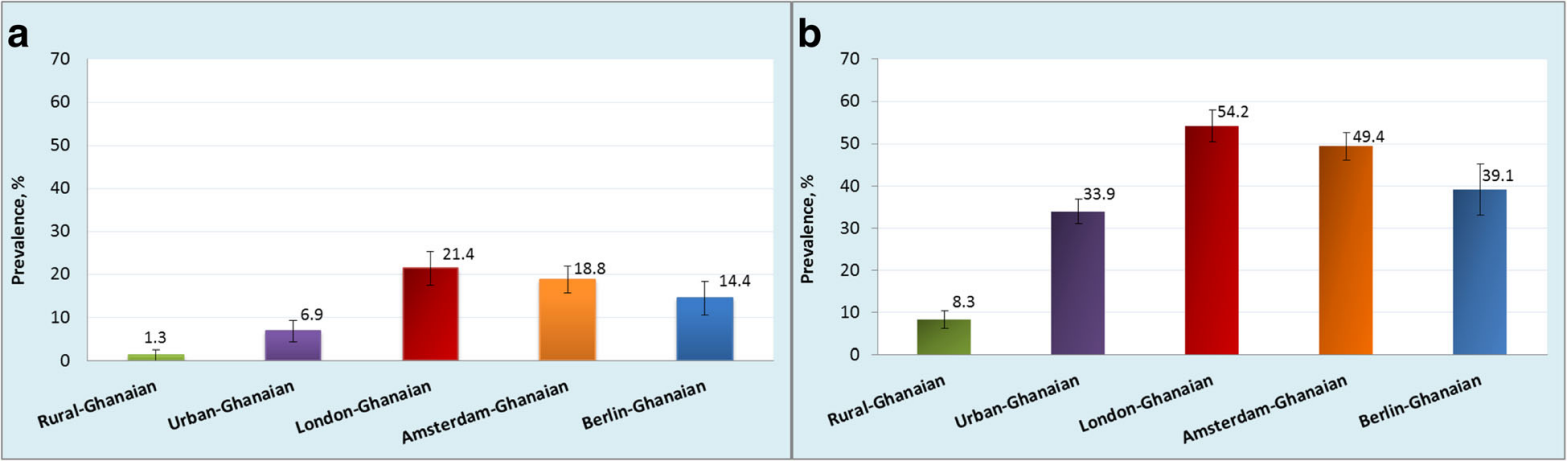
Age-standardised prevalence of obesity (BMI ≥ 30 kg/m^2^) by locality in men (**a**) and women (**b**). Error bars are 95 % confidence intervals

**Fig. 2:**
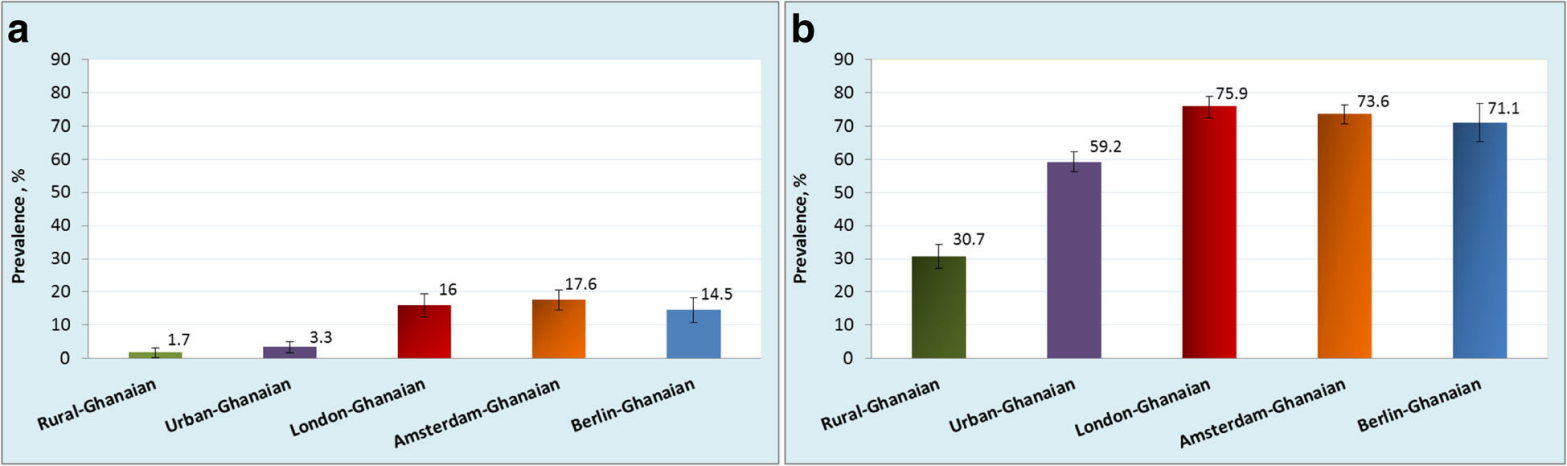
Age-standardised prevalence of abdominal obesity (waist circumference, men: > 102 cm, women: > 88 cm) by locality in men (**a**) and women (**b**). Error bars are 95 % confidence intervals

**Fig. 3:**
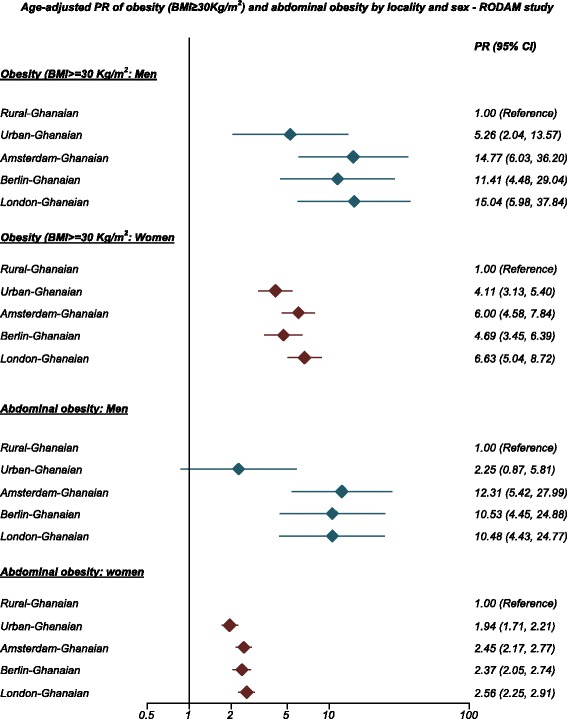
Prevalence ratios of obesity (BMI ≥ 30 kg/m^2^) and abdominal obesity by locality and sex (models are adjusted for age and education)

The age-standardised prevalence of generalised obesity varied between the five population groups, ranging from 1 % in rural Ghana to 21 % in London in men, and from 8 % in rural Ghana to 54 % in London in women (Fig. 1a, b). Similar large differences were also observed for abdominal obesity with the age-standardised prevalence rates in men, ranging from 2 % in rural Ghana to 18 % in Amsterdam, and in women from 31 % in rural Ghana to 76 % in London (Fig. 2a, b). The differences between individuals living in rural Ghana and Ghanaians living in different sites increased with age for both generalised obesity (Additional file 2: Figure S2) and abdominal obesity (Additional file 3: Figure S3). Figure 3 illustrates adjusted PRs for obesity using rural Ghanaians as the reference category. For men, the PR of obesity was five times higher in urban Ghanaians than in their rural counterparts. Among Ghanaian men living in Europe, the obesity PR increased 11- to 15-fold across all cities. For women, the PR of obesity was four times higher in urban Ghanaians than in rural dwellers. As for European cities, obesity PR in Ghanaian women was increased up to 6.6-fold in London. Similarly, the adjusted PRs of abdominal obesity were higher in all sites than in rural Ghana, although the difference between men in rural and urban Ghana was statistically non-significant (Fig. 3).

When overweight and obesity were combined, 8 % of men in rural Ghana were considered overweight or obese, whereas this figure was nearly 40 % in urban Ghana, 64 % in Berlin, 68 % in Amsterdam, and 75 % in London (Additional file 4: Figure S4). In women, this proportion was high even in rural Ghana (34 %), and 69 % in urban Ghana and 80–90 % in European cities (Additional file 4: Figure S4).

### Prevalence of type 2 diabetes and IFG

**Fig. 4:**
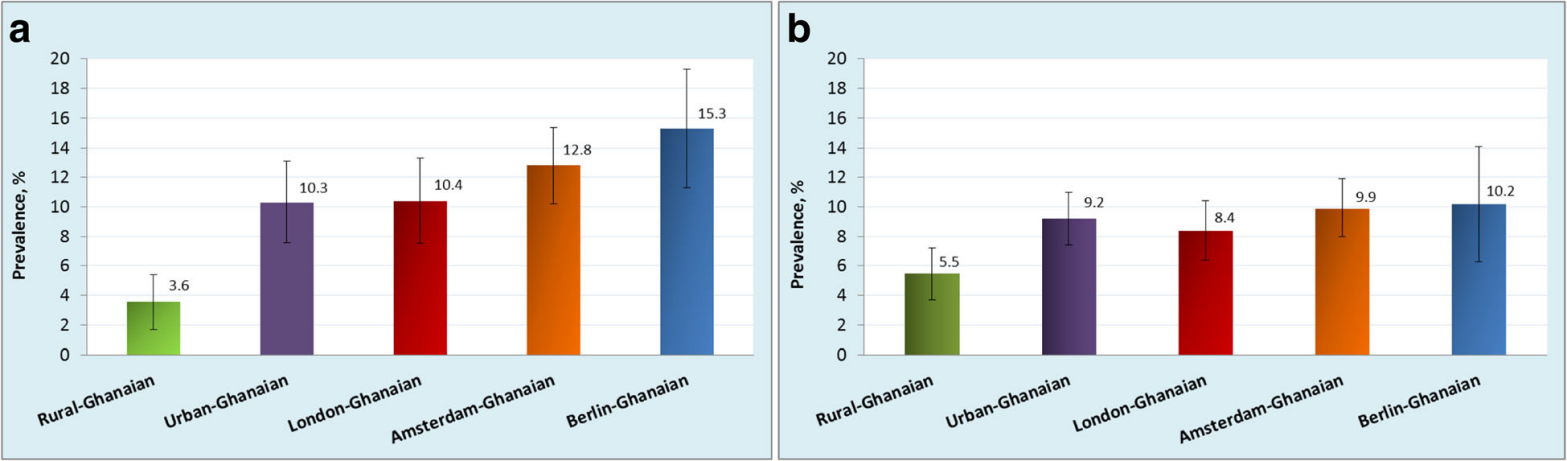
Age-standardised prevalence of type 2 diabetes by locality in men (**a**) and women (**b**). Error bars are 95 % confidence intervals

**Fig. 5:**
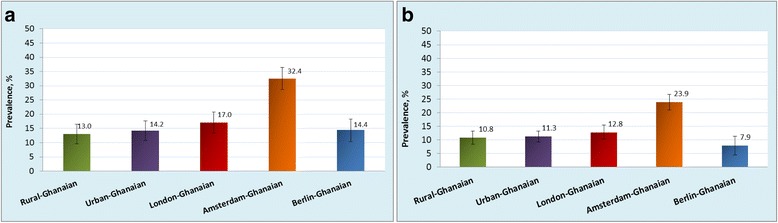
Age-standardised prevalence of impaired fasting glucose by locality in men (**a**) and women (**b**). Error bars are 95 % confidence intervals

**Fig. 6:**
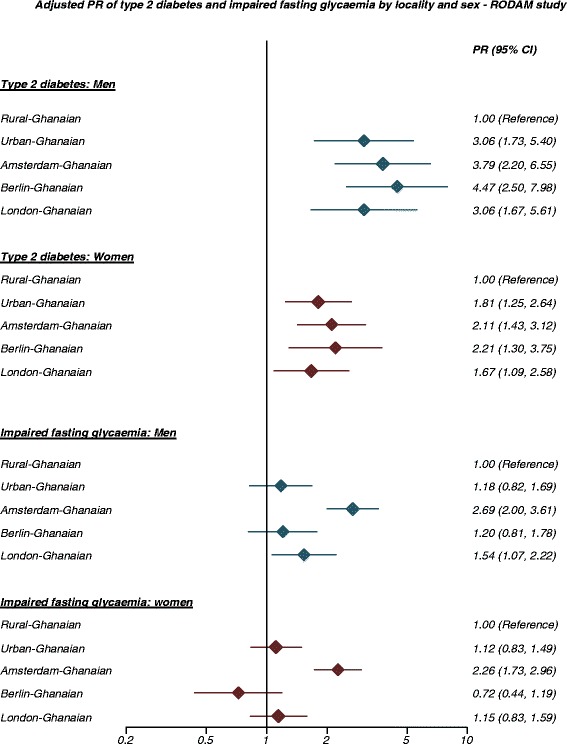
Prevalence ratio of type 2 diabetes and impaired fasting glycaemia by locality and sex (models adjusted for age and education)

The age-standardised prevalence of type 2 diabetes in men and women was 4 % and 6 %, respectively, in rural Ghana (Fig. 4a, b). This proportion was higher in urban Ghana (men, 10 %; women, 9 %) and in Europe, reaching its maximum in Berlin (men, 15 %; women, 10 %). The group-related differences increased with advancing age (Additional file 5: Figure S5). Newly detected type 2 diabetes was more common in urban Ghana than in other sites (Table 1). In addition, we observed a large proportion of the participants with IFG in all sites including rural Ghana (men, 13 %; women, 11 %; Fig. 5a, b). The prevalence of IFG was particularly high in Amsterdam (men, 32 %; women, 24 %) and significantly higher than elsewhere. When rural Ghana was used as the reference category, the PRs of type 2 diabetes in men were nearly 3-fold higher in urban Ghana and increased to nearly 4.5-fold higher in Berlin. In women, the PRs were 1.6-fold higher in London to 2-fold higher in the other sites (Fig. 6).

**Fig. 7:**
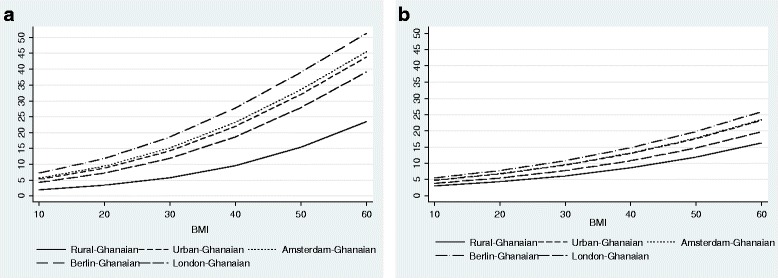
Probability of type 2 diabetes by BMI in men (**a**) and women (**b**) (models are adjusted for age)

**Fig. 8:**
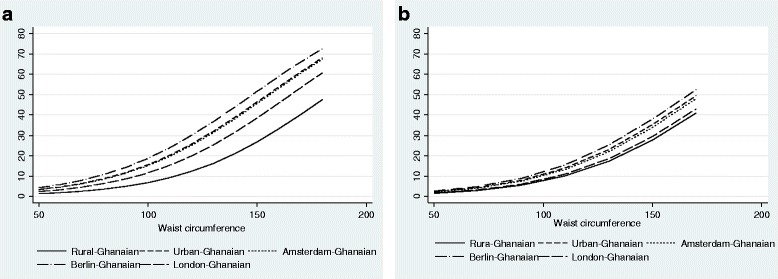
Probability of type 2 diabetes by waist circumference in men (**a**) and women (**b**) (models are adjusted for age)

The probability of type 2 diabetes increased with high levels of BMI (Fig. 7a, b) and waist circumference (Fig. 8a, b). However, with a given level of BMI and waist circumference, the probability of type 2 diabetes was greater among urban and migrant Ghanaians than their rural Ghanaian peers in both men and women, although the magnitude of the differences were greater in men than in women.

## Discussion

### Key findings

Our findings from a large, multi-centre and multi-country study show higher rates of obesity and type 2 diabetes in urban Ghanaians and Ghanaian migrants in Europe than in rural Ghana. For obesity, there was a clear rising gradient in prevalence from rural through urban Ghana to migrants in Europe. No such gradient was observed for type 2 diabetes and IFG across the sites. Notably, the difference in type 2 diabetes prevalence between urban Ghana and Ghanaians in Europe was rather small. The prevalence of IFG was generally high and similar across sites except for the even higher prevalence in Amsterdam.

### Strengths and limitations

The main strength of the RODAM study is the use of well-standardised approaches across the various study sites. Another unique strength of this study is the homogenous study population of Ghanaians living in different settings in Africa and Europe. So far, only a few studies have attempted to assess the potential role of migration on obesity and type 2 diabetes among African populations by comparing native Africans with people of African ancestry living in the Caribbean, UK and USA [11, 12]. However, these studies were limited due to the heterogeneous ancestry of populations who were transported out of Africa several centuries ago. This factor, as well as genetic admixture primarily with European ancestry population groups, make it difficult to assess the potential role of migration and its impact on health in African populations [20]. Furthermore, these studies were based on secondary data with different measurement protocols. The RODAM study overcomes these previous limitations by focusing on one population using the same measurement procedures in all sites.

Our study also has limitations. First, as in most epidemiological studies, type 2 diabetes was defined by a single blood glucose measurement, which traditionally would have to be confirmed. Second, although the same methods were applied in all sites, the recruitment strategies had to be adapted to suit the local circumstances due to differences in registration systems. Ghanaian participants in Amsterdam, for example, were drawn from the Amsterdam Municipal Population register, whereas London participants were drawn mainly from Ghanaian organisations lists. It is possible that individuals who were not on the lists of these organisations differ in terms of sociodemographics, which might somewhat affect the representativeness of Ghanaian migrants in London and Berlin. In a non-response analysis, men more often were non-respondents than women in all sites except for Berlin. Non-respondents were younger than respondents in all sites. Further, the non-response analysis in Berlin revealed that the distribution of respondents and non-respondents across Berlin city districts was fairly similar. Additionally, evidence suggests that most Ghanaians in Europe are affiliated with Ghanaian organisations [13, 14], suggesting that members within these organisations may be representative of the Ghanaian population living in various European countries. Therefore, although a certain level of bias is likely, as in all population-based surveys, we consider it unlikely that the differences in prevalence rates between European sites are substantially biased by the variations in sampling strategy. Finally, only fasting plasma glucose was used to diagnose diabetes, which may underestimate the prevalence of diabetes. Evidence suggests that the 2-h plasma glucose value after a 75-g oral glucose tolerance test diagnoses more people with diabetes fasting plasma glucose.

### Discussion of the key findings

Our current findings show that obesity is extremely common among women at all study sites, including a notable prevalence in rural settings. The prevalence rates in men were less than half of those among women. Despite the higher burden among urban populations, overweight/obesity is rapidly increasing also in rural communities in low- and middle-income countries, especially among women, as our study clearly shows. Therefore, the notion that overweight/obesity is affecting typically the urban populations can no longer be substantiated [7, 8]. In fact, over a third of women in rural Ghana were either overweight or obese. This corroborates recent findings in rural South African youth [21]. Rapid urbanisation and improved contact between rural and urban settings due to infrastructure improvements may be facilitating the transfer and introduction of urban practices to rural settings with consequent changes in diet, resulting in consumption of energy-dense traditional or processed foods as seen in urban Ghana and some settings in SSA [22, 23]. Of note, the present study shows that the obesity rate among women in urban Ghana is nearly as high as those reported among women in the USA [24], and far higher than the prevalence rates reported among women in many European high-income countries [25–27]. We show that Ghanaian migrants in Europe are particularly affected by obesity, the rate being up to 15 times higher than among their rural counterparts in Africa. Among migrant Ghanaian women, the obesity rate greatly exceeds the figures of the host European populations in all three European countries. In the 2013 Health Survey for England, the prevalence of obesity among English general population women was 24 % [25] compared with 54 % observed in the present study among Ghanaian migrant women in London. Similarly, the prevalence of obesity among Dutch women is 13 % [26] compared with 49 % in Ghanaian migrant women in Amsterdam, and 24 % in German women [27] compared with 39 % among Ghanaian migrant women in Berlin.

Worryingly, type 2 diabetes occurred at a similar prevalence among individuals in urban Ghana and in Europe. Previous studies among SSA populations found a rising gradient of type 2 diabetes from SSA through the Caribbean to the UK and USA [11, 12]. Mbanya et al. [12], for example, reported an age-standardised prevalence of diabetes of 1 % among urban Cameroonian men compared with 15 % in African Caribbeans in the UK. This gradient was due to extremely low prevalence of type 2 diabetes in SSA, which has been documented from the earliest studies that were conducted more than five decades ago. For example, in a 1958 study, Dodu et al. [28] observed a diabetes prevalence of 0.4 % in an urban population in Accra, Ghana. Likewise, a community-based study in the Volta region of Ghana in 1964 found a diabetes prevalence of 0.2 % [29]. In contrast, the results of the present study suggest that the gradient between urban Africans and diaspora African living in high-income European countries is fading rapidly. In fact, the prevalence of type 2 diabetes among women was marginally higher in urban Ghana (9.2 %) than in London (8.4 %). Thus, the increasing risk of type 2 diabetes is no longer an issue of only migrant populations, but appears to have reached urban communities in SSA. This implies increased risks for rural African communities, especially given the rapid changing lifestyles in these settings. The rise of obesity and type 2 diabetes among SSA populations can be partly attributed to modernisation with consequent adoption of unhealthy aspects of globalised lifestyles such as physical inactivity and poor dietary behaviour [22]. The key specific drivers within these broad categories, however, still need to be identified. Interestingly, the prevalence of type 2 diabetes was higher in men than in women despite the higher levels of obesity in women in all sites except rural Ghana. The explanations for these differences are unclear, but may be partly due to a more favourable body fat distribution in women [30]. Alternatively, it is possible that body weight has a larger impact on type 2 diabetes risk among men than among women, as is suggested by the current study.

Another important finding from this study is the high prevalence of IFG in all sites. The IFG rates in both rural (12 %) and urban (13 %) Ghana are far higher than those in most urban populations in Africa [31, 32]. In a community-based study conducted more than a decade ago in urban Accra, the IFG prevalence was 6.2 % [33], indicating a nearly 110 % percentage increase in IFG in urban Ghana in a decade. In the present study, IFG was exceptionally common in Amsterdam Ghanaian migrants, which is consistent with our earlier findings [10, 34, 35]. In a previous study, the prevalence of IFG was 35 % and 14 % among African Caribbeans in the Netherlands and in England, respectively [34]. The high rate of IFG is worrying given the increased risk of developing type 2 diabetes and related complications [36]. The reasons for the abundance of IFG among Ghanaians in the Netherlands is unclear but might be due to contextual factors such as differences in treatment of diabetes and/or unknown aetiological factors; this requires further study.

Despite varying prevalence rates among the host populations in the three European countries, the respective differences among migrants residing in these countries were rather small. Still, although higher, the type 2 diabetes prevalence among the migrant populations mimics their respective host European populations. Recent International Diabetes Federation age-standardised estimates indicate a prevalence of type 2 diabetes of 4.7 % in the UK, 5.5 % in the Netherlands, and 7.4 % in Germany [1]. Despite the lower prevalence of type 2 diabetes in the UK [25], obesity is more common in the UK than in most European countries [26, 27]. Interestingly, a similar pattern was observed among our study populations with Ghanaians in London having a lower prevalence of type 2 diabetes but a higher prevalence of obesity compared with Amsterdam and Berlin. This observation seems to suggest that the national contextual factors, such as prevailing health behaviour, health-related policies and access to preventive services, may influence metabolic risk factors in different ways in various countries [6, 37].

Our findings have important public health implications for health planners in Europe and Africa. The prevalence rates of obesity and type 2 diabetes among African migrants exceed those of the European host populations. Ghana is a lower middle-income country with a substantial burden of communicable diseases. The high levels of overweight and type 2 diabetes will undoubtedly put more pressure on the already overburdened health system suggesting an urgent need for action with strong support by government and civil societies in Ghana. This requires a health policy shift towards prevention and control of obesity and diabetes and other non-communicable diseases [38].

## Conclusions

Our study findings show that obesity, IFG and type 2 diabetes are common in both SSA migrants and their population of origin. The findings show a gradient of rising prevalence from rural through urban Africa to Europe for obesity, but not for the type 2 diabetes gradient between urban African and Europe, which has reached almost European levels in urban Ghana. This seems to suggest that the increased risk of type 2 diabetes is no longer limited to migrant populations, and that processes similar to those underlying the high burden in migrants may also be at play in SSA, particularly in urban centres. This, in turn, points to an urgent need to unravel the potential factors contributing to the high prevalence of these conditions in both SSA migrants and non-migrants to inform targeted intervention and prevention programmes.

## Additional files

Additional file 1: Figure S1. Flow chart of inclusion of RODAM study participants in analysis. (DOC 40 kb)
Additional file 2: Figure S2. Probability of obesity (BMI≥30kg/m2) by age in men (A) and women (B). (DOC 33 kb)
Additional file 3: Figure S3. Probability of abdominal obesity (waist circumference, men: >102 cm, women: >88 cm) by age in men (A) and women (B). (DOC 34 kb)
Additional file 4: Figure S4. Age-standardised prevalence of overweight (BMI≥25 kg/m2) by locality in men (A) and women (B). Error bars are 95% confidence intervals. (DOC 137 kb)
Additional file 5: Figure S5. Probability of type 2 diabetes by age in men (A) and women (B). (DOC 34 kb )

## Abbreviations

BMI: body mass index
IFG: impaired fasting glycaemia
PR: prevalence
RODAM: Research on Obesity & Diabetes among African Migrants
SSA: sub-Saharan Africa
