# Supplementary material for: Obesity and type 2 diabetes in sub-Saharan Africans – Is the burden in today’s Africa similar to African migrants in Europe? The RODAM study
Source: BMC Med. 2016 Oct 21;14:166. doi: 10.1186/s12916-016-0709-0 (PMC5075171; doi:10.1186/s12916-016-0709-0)
Supplement: Additional file 1: Figure S1. — Flow chart of inclusion of RODAM study participants in analysis. (DOC 40 kb) [file 12916_2016_709_MOESM1_ESM.doc]

**Agreed to participate**

**n = 6385**

**Completed physical examination**

**and blood collection**

**n= 5898**

**No attempt for blood collection or did not attend physical examination**

**n= 487**

**Outside age range (25-70)**

**n= 239**

**Included in overall analysis**

**n= 5659**

Figure S1| Flow chart of inclusion of RODAM study participants in analysis
