# Supplementary material for: Obesity and type 2 diabetes in sub-Saharan Africans – Is the burden in today’s Africa similar to African migrants in Europe? The RODAM study
Source: BMC Med. 2016 Oct 21;14:166. doi: 10.1186/s12916-016-0709-0 (PMC5075171; doi:10.1186/s12916-016-0709-0)
Supplement: Additional file 2: Figure S2. — Probability of obesity (BMI≥30kg/m2) by age in men (A) and women (B). (DOC 33 kb) [file 12916_2016_709_MOESM2_ESM.doc]

Figure S2| Probability of obesity (BMI≥30kg/m^2^) by age in men (A) and women (B)
