# Supplementary material for: Obesity and type 2 diabetes in sub-Saharan Africans – Is the burden in today’s Africa similar to African migrants in Europe? The RODAM study
Source: BMC Med. 2016 Oct 21;14:166. doi: 10.1186/s12916-016-0709-0 (PMC5075171; doi:10.1186/s12916-016-0709-0)
Supplement: Additional file 4: Figure S4. — Age-standardised prevalence of overweight (BMI≥25 kg/m2) by locality in men (A) and women (B). Error bars are 95% confidence intervals. (DOC 137 kb) [file 12916_2016_709_MOESM4_ESM.doc]

**
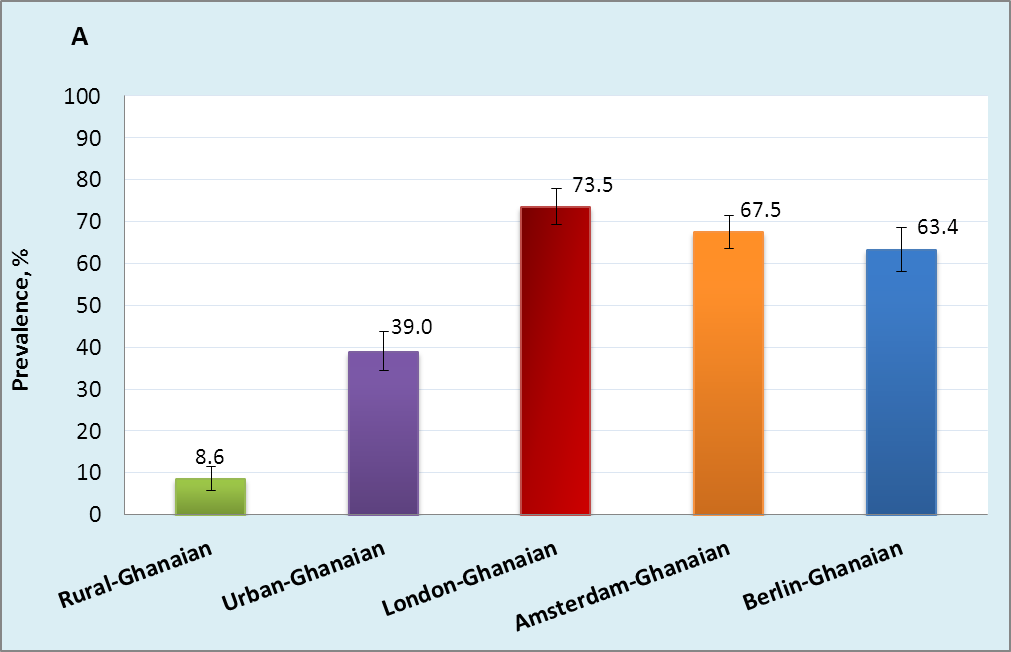

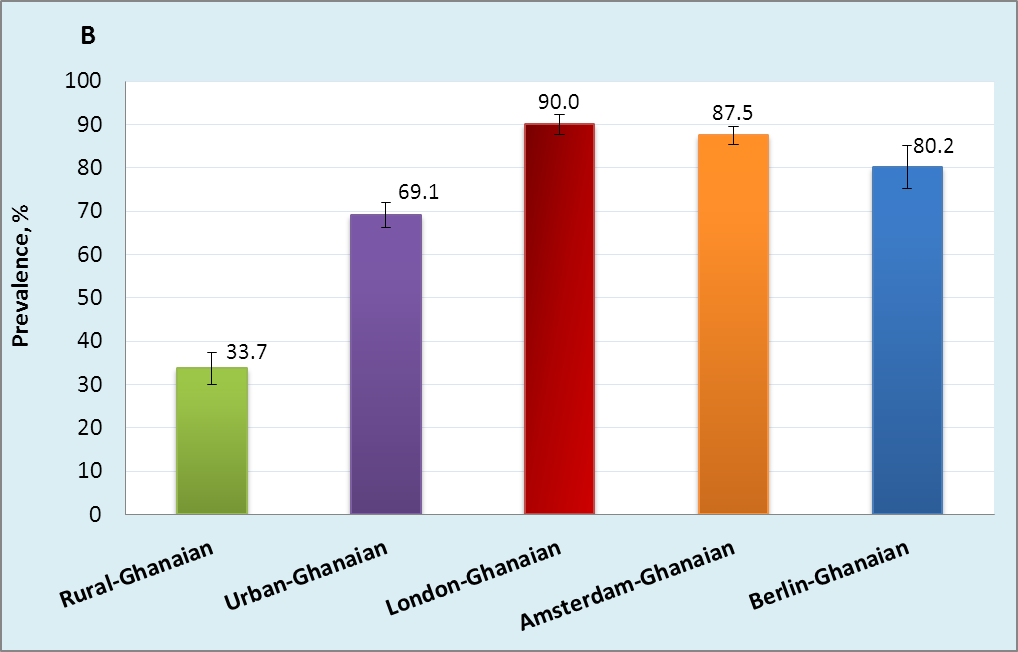
**

Figure S4| Age-standardised prevalence of overweight (BMI≥25 kg/m^2^) by locality in men (A) and women (B). Error bars are 95% confidence intervals.
