# Supplementary material for: Obesity and type 2 diabetes in sub-Saharan Africans – Is the burden in today’s Africa similar to African migrants in Europe? The RODAM study
Source: BMC Med. 2016 Oct 21;14:166. doi: 10.1186/s12916-016-0709-0 (PMC5075171; doi:10.1186/s12916-016-0709-0)
Supplement: Additional file 5: Figure S5. — Probability of type 2 diabetes by age in men (A) and women (B). (DOC 34 kb ) [file 12916_2016_709_MOESM5_ESM.doc]

Figure S5| Probability of type 2 diabetes by age in men (A) and women (B)
